# Psychological burden in anaphylaxis: a study on alexithymia and quality of life

**DOI:** 10.3389/falgy.2026.1791020

**Published:** 2026-07-06

**Authors:** Vincenzo Patella, Luciana Pierro, Tiziana Peduto, Ottavia Inglese, Giovanni Florio, Ada Giuliano, Roberta Zunno

**Affiliations:** 1 Department of Internal Medicine and Division of Allergy and Clinical Immunology ASL Salerno, “Santa Maria Della Speranza” Hospital, Battipaglia, Italy; 2 Postgraduate Program in Allergy and Clinical Immunology, University of Naples Federico II, Naples, Italy; 3 Simple Departmental Operating Units of Tossicology Laboratory, ASL Salerno, Salerno, Italy

**Keywords:** alexithymia, anaphylaxis, psychological burden, quality of life, stress

## Abstract

**Aim:**

This study examines the impact of psychological burden on quality of life (QoL) in patients with anaphylaxis, assessing the relationship between alexithymia, QoL, and the presence of anxiety or depression.

**Materials and methods:**

A total of 29 patients with a confirmed diagnosis of anaphylaxis and a prescription for self-injectable epinephrine were recruited. Their QoL, psychological stress levels, and potential presence of anxiety, depression, and alexithymia were assessed using validated questionnaires: The Anaphylaxis Quality of Life Scale for Adults (A-QoL-Adults), the World Health Organization Quality of Life Scale – Brief Version (WHOQoL-BREF), the Perceived Stress Scale (PSS), the Hospital Anxiety and Depression Scale (HADS-A and HADS-D), and the Toronto Alexithymia Scale (TAS-20). Scores were compared, and statistical analyses were conducted.

**Results:**

Of the 29 patients who completed the questionnaires, 11 (37.9%) were classified as alexithymic or borderline alexithymic. A moderate positive correlation was observed between TAS-20 scores and perceived anaphylaxis severity (r = 0.435, *p* = 0.021), with alexithymic/borderline alexithymic patients reporting significantly higher severity compared to non-alexithymic patients (high severity: 35.3% non-alexithymic vs. 81.8% alexithymic/borderline alexithymic, *p* = 0.016). TAS-20 scores showed a strong positive correlation with HADS-D scores (r = 0.518, *p* = 0.006) and a strong negative correlation with Environmental-WHOQoL-BREF scores (r = −0.501, *p* = 0.008). Additionally, moderate positive correlations were found between TAS-20 scores and HADS-A (r = 0.408, *p* = 0.035), A-QoL-Adults (r = 0.487, *p* = 0.009), and its subscales SQoL (r = 0.477, *p* = 0.01) and LoL (r = 0.479, *p* = 0.01). Finally, TAS-20 scores exhibited a moderate negative correlation with Psychological-WHOQoL-BREF scores (r = −0.501, *p* = 0.008).

**Conclusion:**

This study underscores the significant correlation between alexithymia and QoL in patients with anaphylaxis. Integrating psychological assessments into anaphylaxis management may enable healthcare providers to develop more comprehensive strategies beyond physical symptom control. A holistic approach—including education, emotional support, and psychological interventions—could enhance long-term outcomes for patients prescribed epinephrine auto-injectors.

## Introduction

Anaphylaxis is a severe allergic reaction that can significantly impact patients' quality of life (QoL) and shape their emotional experiences, affecting both physical and mental health. In particular, it can impose a psychological burden, sometimes associated with depression, anxiety, and post-traumatic stress disorder (PTSD) (1–5).

Moreover, the constant fear of anaphylaxis can have a detrimental effect on mental health, not only for patients but also for their caregivers. This fear can interfere with daily life, manifesting in behaviours such as allergen avoidance, social limitations or isolation, heightened stress about safety, and a perceived restriction of personal freedom (4–10).

Recent studies emphasize the importance of addressing the psychological burden in patients with anaphylaxis and their caregivers, advocating for a patient-centered, multidisciplinary approach that includes psychological counseling (11, 12).

Few studies have examined the correlation between anaphylaxis and alexithymia (13–16), a psycho-affective dysfunction characterized by an impaired ability to identify, describe, and express one's own emotions and those of others. Individuals with alexithymia struggle to distinguish emotional states from physical sensations, exhibit hypersensitivity to physical stimuli, have limited imagination, and tend toward concrete thinking. These traits can lead to increased avoidance behaviors in fear-inducing situations, such as the risk of anaphylaxis, ultimately exacerbating mental distress (17, 18).

Alexithymia is marked by a weak connection between the cognitive-experiential level of emotions (subjective awareness and verbal expression of affective states), the physiological level (involving the amygdala, hypothalamus, autonomic nervous system, and neuroendocrine activation), and the motor-expressive level (e.g., facial expressions, posture, and vocal tone) (17, 18).

Alexithymic individuals are at greater risk of developing chronic conditions such as atopic dermatitis, as their difficulties in stress management and emotional regulation negatively impact neuroendocrine and immune system function (19–21).

In our recent article, we conducted a pilot study to develop and validate an Italian-language version of the Anaphylaxis Quality of Life Scale for Adults (A-QoL-Adults) (22). This adaptation aims to guide and monitor appropriate interventions to enhance treatment adherence and improve the quality of life (QoL) of patients prescribed epinephrine auto-injectors.

In this study, we aim to consider the impact of psychological burden on QoL by evaluating the relationship between alexithymia and QoL in patients with anaphylaxis.

## Material and methods

29 patients with a confirmed diagnosis of anaphylaxis and prescription of self-injectable epinephrine were recruited between September 2022 and April 2023 at the Allergy and Clinical Immunology outpatient clinic of the “Santa Maria della Speranza” Hospital in Battipaglia. The following information about their anaphylaxis was collected using a structured questionnaire administered by an allergist specialist: the cause of anaphylaxis (food, drugs, Hymenoptera, latex, or spontaneous anaphylaxis), the time since the diagnosis of anaphylaxis (expressed in months), a self-assessment of the severity of anaphylaxis (using a Likert scale from 1 to 10, distinguishing values from 0 to 8 as “mild-moderate severity” and those from 9 to 10 as “high severity”). During the outpatient visit, a psychology specialist also administered the following questionnaires to the patients: The A-QoL-Adults, with its subscales Emotional Impact (EQoL), Social Impact (SQoL), and Limitations on Life (LoL); The “World Health Organization Quality of Life Scale” – Brief Version (WHOQoL BREF), divided into its four domains Physical QoL, Psychological QoL, Social QoL, Environmental QoL; the Hospital Anxiety and Depression Scale (HADS) for the evaluation of anxiety (HADS-A) and depression (HADS-D); The Perceived Stress Scale (PSS) that measure the psychological stress levels; The Toronto Alexithymia Scale (TAS-20), including its three domains difficulty identifying feelings (DIF), difficulty communicating and describing feelings (DDF), and external-oriented thinking (EOT), for the diagnosis of alexithymia. The questionnaires are described in detail in Table 1. In this study, patients with a TAS-20 score >51, including borderline values (52–60) that indicate possible alexithymia, were considered into the same group of alexithymic patients, named alexithymic/borderline alexithymic.

**Table 1:** Description and scoring system of the questionnaires used in this study.

The Anaphylaxis Quality of Life Scale for Adults (A-QoL-Adults) (23) is a 28-item series of questions to be administered to adults with anaphylaxis from any cause, with a 1-to-5 response scale (1 = never, 2 = rarely, 3 = sometimes, 4 = most of the time, and 5 = always). It was recently developed by Knibb et al. in 2022 and measures the impact of anaphylaxis on patients' quality of life. It has three subscales: Emotional Impact (EQoL), Social Impact (SQoL), and Limitations on Life (LoL). Higher scores indicate worse anaphylaxis-related quality of life. A recent article (22) validated an Italian version of the A-Qol-adults questionnaire, used in this study.
The “World Health Organization Quality of Life Scale” – Brief Version (WHOQoL BREF) (24, 25) is a 26-item version of the WHOQOL-100 assessment, with a 1-to-5 response scale, which has proven to be a sound, cross-culturally valid assessment of patients' QoL. The questionnaire analyzes four domains: Physical QoL, Psychological QoL, Social QoL, and Environmental QoL. The four domain scores are each converted into a scale from 0 to 100, where 0 points represent the worst possible state of health, while 100 points represent the best possible state of health about the respective domain. Therefore, the lower the final score, the greater the limitations or problems and the worse the quality of life. This study used the Italian-validated version (26).
The Hospital Anxiety and Depression Scale (HADS) (27) is a self-administer standardized questionnaire capable of identifying and quantifying states of anxiety and depression in patients with organic diseases. It comprises two 7-item scales, one for evaluating anxiety (HADS-A) and the other for assessing depression (HADS-D), with a 0-to-3 response scale for each item, for a total of 21 points for each domain. Higher scores indicate worse states of anxiety or depression. In particular, 0-7 scores are considered normal, from 8 to 10 indicate borderline situations, and scores greater than 11 indicate clinically relevant anxiety or depression. This study used the Italian validated version (28).
The Perceived Stress Scale (PSS) (29) is a 10-item questionnaire widely used to measure individual psychological stress levels. The 10 questions ask about patients' feelings and thoughts during the last month with a 0-to-4 response scale (0 = never, 1 = almost never, 2 = sometimes, 3 = fairly often, 4 = very often). Therefore, Individual scores on the PSS can range from 0 to 40, with higher scores indicating higher perceived stress.
The Toronto Alexithymia Scale (TAS-20) (30, 31) is a 20-item questionnaire on the evaluation of alexithymia. The TAS-20 has 3 subscales: Difficulty Describing Feelings (DDF) subscale, related to 5 items; Difficulty Identifying Feeling (DIF) subscale, related to 7 items; Externally-Oriented Thinking (EOT) subscale, used to measure the tendency of individuals to focus their attention externally and related to 8 items. Items have a 1-to-5 response scale (1 = strongly disagree and 5 = strongly agree). The total alexithymia score is the sum of responses to all 20 items, while the score for each subscale factor is the sum of the responses to that subscale. Therefore, higher scores indicate a worse alexithymia condition. The cut-off is: score equal to or lower than 51 = non-alexithymia; 52-60 = borderline, that is, with possible alexithymia; equal to or greater than 61 = alexithymia.

The values thus obtained were entered into a database, and statistical analysis was performed using SPSS. Categorical data were indicated as numbers and percentages. The distribution of continuous variables was determined by Kolmogorov–Smirnov Test. Non-normally distributed, continuous variables were shown as median (Q1–Q3). Mann–Whitney-U non-parametric test and Pearson Chi-Square were used to compare the differences between alexithymic/borderline alexithymic group and non-alexithymic group regarding gender, age, time since the diagnosis of anaphylaxis in months, anaphylaxis severity, and the other questionnaires scores. TAS-20 was compared to the other questionnaires using Spearman's rank correlation coefficient: a value greater than 0.5 indicates a strong correlation; from 0.3 to 0.49, a medium correlation; less than 0.29 indicates a weak correlation. A value of *p* < 0.05 was considered statistically significant.

## Results

The characteristics of the studied population are summarized in Table 2. 29 patients participated in the study, 13 males (44.8%) and 16 females (55.2%), with a median age of 38 (23.5–52) years and a median time since the diagnosis of anaphylaxis of 14 (8.5–31) months. Regarding the causes of anaphylaxis, they were mainly foods (13 cases, 44.8%) and Hymenoptera (6 patients, 20.7%), while in 3 cases, it was latex (10.3%), in 2 cases drugs (6.9%) and in the remaining 5 cases it was spontaneous anaphylaxis or of unknown cause (17.3%). 16 patients (55.2%) considered their anaphylaxis to be highly severe.

**Table 2:** Demographic and anaphylaxis characteristics of the sample and questionnaires scores.

Population characteristics	*n* = 29
Median age, y	38 (23.5–52)
Age range, y	21–70
Male gender	13 (44.8%)
Female gender	16 (55.2%)
Cause of anaphylaxis	
Hymenoptera	6 (20.7%)
Food	13 (44.8%)
Drugs	2 (6.9%)
Latex	3 (10.3%)
Unknown	5 (17.3%)
Symptoms severity	
Median	9 (6–10)
Mild-moderate severity (1-8), n.	13 (44.8%)
High severity (9-10), n.	16 (55.2%)
Non-alexithymic	18 (62.1%)
Alexithymic/borderline alexithymic	11 (37.9%)
Questionnaires scores (median)	
TAS-20	45 (33.5–57.5)
DIF	15 (10–23)
DDF	13.5 (8.25–16.75)
EOT	17 (13–22)
HADS-A	9 (4.5–14)
HADS-D	4 (2.5–8)
PSS	17 (11–23.5)
A-Qol-Adults	2.05 (1.42–3.57)
SQoL	1.67 (1.11–3.16)
EQoL	2.5 (1.58–4.33)
LoL	2.5 (1.58–3.5)
WHOQoL BREF	
Physical-QoL	67.86 (55.35–78.57)
Psychological-QoL	62.5 (45.83–70.83)
Social-QoL	66.67 (50–75)
Environmental-QoL	62.5 (51.56–81.24)

Of the 29 patients who completed the questionnaires, 11 were alexithymic/borderline alexithymic (37.9%). The median TAS-20 score was 45 (33.5–57.5), while for the subscales, the median DIF score was 15 (10–23), the median DDF score was 13.5 (8.25–16.75), and the median EOT score was 17 (13–22). The scores of the other questionnaires are summarized in Table 2.

Moreover, anaphylactic patients with high severity of symptoms had a significant higher HADS-A score (11 vs. 7; Mann–Whitney-U: U = 64.500, *p* = 0.022), A-QoL-Adults (3.09 vs. 1.76; Mann–Whitney-U: U = 55.000, *p* = 0.031), SQoL (2.72 vs. 1.22; Mann–Whitney-U: U = 56.500, *p* = 0.036), a significant lower Physical-WHOQoL BREF (60.71 vs. 69.64; Mann–Whitney-U: U = 49.000, *p* = 0.027) and a particularly significant lower Psychological-WHOQoL Bref (47.91 vs. 66.67; Mann–Whitney-U: U = 34.500, *p* = 0.004) than patients with mild-moderate symptoms (Table 3). The differences between HADS-D scores were not significant.

**Table 3:** Differences between anaphylactic patients with mild to moderate severity of symptoms and high severity of symptoms.

Variable considered	Mild-moderate severity anaphylaxis (*n* = 13)	High severity of anaphylaxis (*n* = 16)	*p*-value
TAS-20	**35 (27–47.5)**	**54 (43–59)**	**0.024**
TAS-20 DIF	**11 (9–15.5)**	**22 (12–25)**	**0.022**
TAS-20 DDF	**11 (5–15)**	**16 (11–20)**	**0.023**
TAS-20 EOT	**16 (12.5–20.5)**	**19 (13–23)**	**0.277**
PSS	15.5 (11–21.5)	22 (11.5–26)	0.143
HADS-A	**7 (4–10)**	**11 (7.25–14.75)**	**0.022**
HADS-D	3 (2.25–6.75)	5 (3.25–10)	0.141
Physical-WHOQoL BREF	**69.64 (67.86–78.57)**	**60.71 (47.32–73.21)**	**0.027**
Psychological-WHOQoL BREF	**66.67 (62.50–73.95)**	**47.91 (33.33–64.58)**	**0.004**
Social-WHOQoL BREF	66.67 (58.33–81.24)	58.33 (50–72.91)	0.224
Environmental-WHOQoL BREF	64.06 (59.38–76.56)	60.94 (45.31–82.81)	0.339
A-QoL-Adults	**1.76 (1.21–2.45)**	**3.09 (1.96–4.04)**	**0.031**
SQoL	**1.22 (1.05–1.78)**	**2.72 (1.35–3.67)**	**0.036**
EqoL	2 (1.50–3.58)	3.16 (2.33–4.45)	0.071
LoL	2 (1.25–2.91)	2.83 (2.21–4.29)	0.062

Questionnaire scores are expressed as median (Q1–Q3).

Values ​​in bold indicate statistical significance (*p* < 0.05).

There were no statistically significant differences between alexithymic/borderline alexithymic and non-alexithymic concerning gender, age, or causes of anaphylaxis, and there were no significant correlations between TAS-20 scores and age, gender, or causes of anaphylaxis. Conversely, there was a medium positive correlation between TAS-20 and anaphylaxis severity in a statistically significant way (Spearman's correlation: *ρ* = 0,435, *p* = 0.021) and a significant difference in anaphylaxis severity in alexithymic/borderline alexithymic compared to non-alexithymic (high severity: 35.3% non-alexithymic vs. 81.8% alexithymic/borderline alexithymic; Pearson Chi-square: *χ*^2^ = 5.812, df = 1, *p* = 0.016; Table 3). Furthermore, patients who perceived a greater severity of anaphylaxis had a statistically significantly higher TAS-20 score (54 vs. 35; Mann–Whitney-U: U = 48.500, *p* = 0.024), DIF score (22 vs. 11; Mann–Whitney-U: U = 48.000, *p* = 0.022) and DDF score (16 vs. 11; Mann–Whitney-U: U = 48.500, *p* = 0.023). This was not the case for the EOT score, where the values were similar.

**Figure 1:**
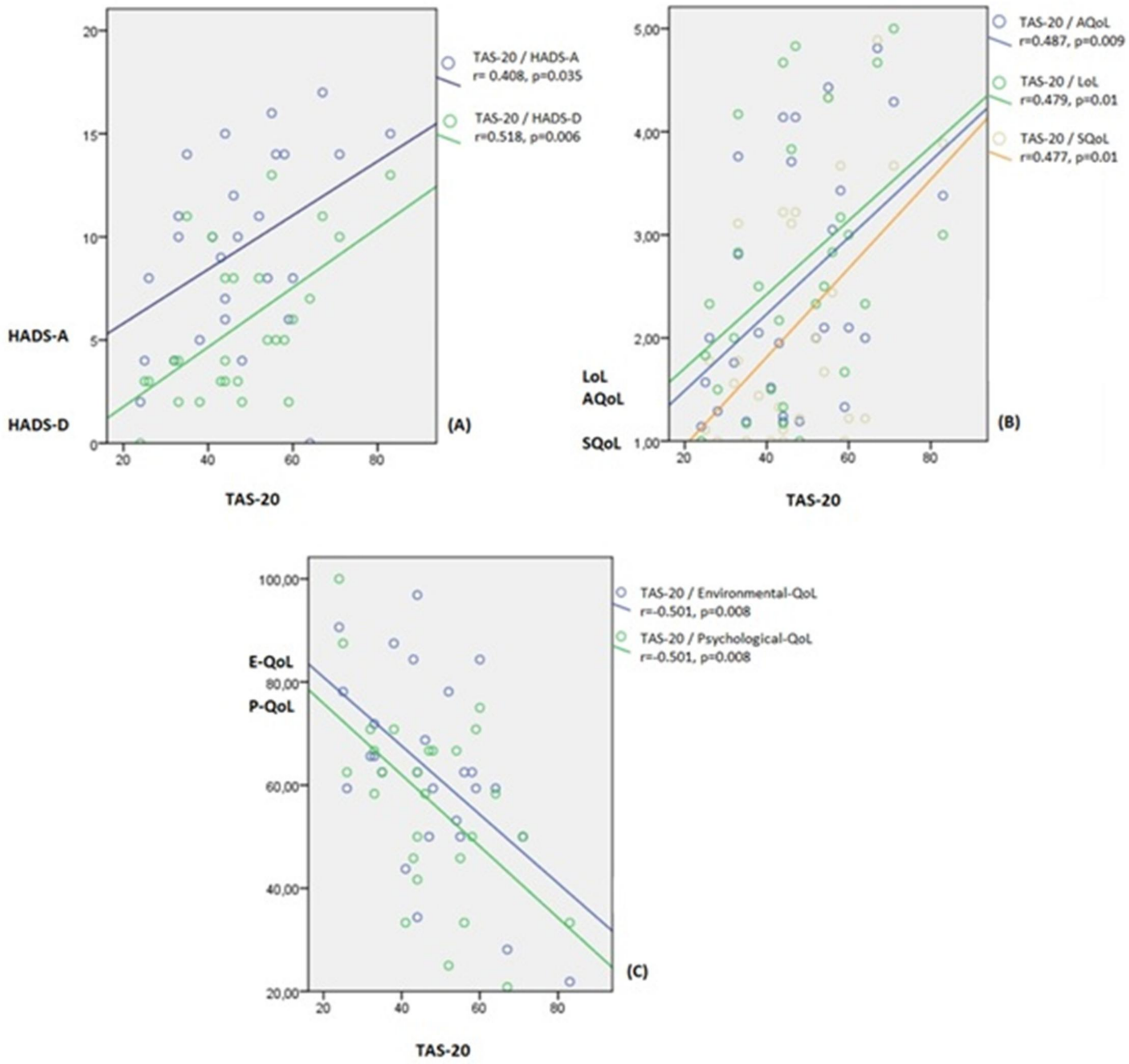
The correlations between TAS-20 and other questionnaires. **(A)** TAS-20 with HADS-A and HADS-D; **(B)** TAS-20 with A-QoL and its subclasses, SQoL and LoL; **(C)** TAS-20 with Environmentale-WHOQoL and Psychological-QHOQoL.

TAS-20 score had a strong positive correlation with HADS-D score (Spearman's correlation: *ρ* = 0.518, *p* = 0.006) and a strong negative correlation with Environmental-WHOQoL BREF score (Spearman's correlation: *ρ* = −0.501, *p* = 0.008); a medium positive correlation with HADS-A score (Spearman's correlation: *ρ* = 0.408, *p* = 0.035), AQoL-adults (Spearman's correlation: *ρ* = 0.487, *p* = 0.009) and its subscales SQoL (Spearman's correlation: *ρ* = 0.477, *p* = 0.01) and LoL (Spearman's correlation: *ρ* = 0.479, *p* = 0.01); finally, TAS-20 score had a medium negative correlation with Psychological-WHOQoL BREF score (Spearman's correlation: *ρ* = −0.501, *p* = 0.008). The correlations are shown in Figure 1.

There were significant differences between alexithymic/borderline alexithymic and non-alexithymic (Table 4) in HADS-D score (7 vs. 3; Mann–Whitney-U: U = 38.500, *p* = 0.014), A-QoL-adults score (3.05 vs. 1.76; Mann–Whitney-U: U = 46.000, *p* = 0.025) and its subscales SQoL score (2.44 vs. 1.33; Mann–Whitney-U: U = 47.500, *p* = 0.029) and LoL score (3 vs. 2; Mann–Whitney-U: U = 48.500, *p* = 0.034).

**Table 4:** Differences between alexithymic/borderline alexithymic and non-alexythimic patients with anaphylaxis.

Variable considered	Alexithymic/borderline alexithymic (*n* = 11)	Non-alexythimic (*n* = 18)	*p*-value
High severity of anaphylaxis	**81.8%**	**35.3%**	**0.016**
PSS	23 (15–28)	15.5 (11–22)	0.079
HADS-A	14 (8–15)	8.5 (4.25–10.75)	0.075
HADS-D	**7 (5–11)**	**3 (2.25–7)**	**0.014**
Physical-WHOQoL BREF	67.86 (50–67.86)	67.86 (61.6–77.68)	0.304
Psychological-WHOQoL BREF	50 (33.33–66.67)	62.5 (52.08–69.79)	0.083
Social-WHOQoL BREF	58.33 (50–66.67)	66.67 (58.33–81.25)	0.149
Environmental-WHOQoL BREF	59.38 (50–62.5)	65.63 (59.38–82.82)	0.071
A-QoL-Adults	**3.05 (2–4.29)**	**1.76 (1.21–3.26)**	**0.025**
SQoL	**2.44 (1.22–3.89)**	**1.33 (1–2.44)**	**0.029**
EqoL	3 (2.33–4.5)	2 (1.5–4.33)	0.164
LoL	**3 (2.33–4.33)**	**2 (1.25–3.33)**	**0.034**

Questionnaire scores are expressed as median (Q1–Q3).

Values ​​in bold indicate statistical significance (*p* < 0.05).

Analyzing the TAS-20 subdomains, the DIF score had a strong positive correlation with PSS (Spearman's correlation: *ρ* = 0.645, *p* < 0.0001), HADS-A (Spearman's correlation: *ρ* = 0.626, *p* < 0.0001), HADS-D (Spearman's correlation: *ρ* = 0.653, *p* < 0.0001), AQoL-adults (Spearman's correlation: *ρ* = 0.690, *p* < 0.0001) and its subscales SQoL (Spearman's correlation: *ρ* = 0.688, *p* < 0.0001), EqoL (Spearman's correlation: *ρ* = 0.596, *p* = 0.001), LoL (Spearman's correlation: *ρ* = 0.659, *p* < 0.0001); a strong negative correlation with Psychological-QoL score (Spearman's correlation: *ρ* = −0.605, *p* = 0.001) and Environmental-QoL (Spearman's correlation: *ρ* = −0.686, *p* > 0.0001); a medium negative correlation with Physical-QoL (Spearman's correlation: *ρ* = −0.427, *p* = 0.026) and Social-QoL (Spearman's correlation: *ρ* = −0.424, *p* = 0.027).

DDF score had a medium positive correlation with HADS-A (Spearman's correlation: *ρ* = 0.402, *p* = 0.038), HADS-D (Spearman's correlation: *ρ* = 0.400, *p* = 0.039) and AQoL-adults score (Spearman's correlation: *ρ* = 0.389, *p* = 0.041); a strong negative correlation with Environmental-QoL (Spearman's correlation: *ρ* = −0.507, *p* = 0.007); a medium negative correlation with Physicological-QoL score (Spearman's correlation: *ρ* = −0.447, *p* = 0.019).

The EOT score had no statistically significant correlation with the other questionnaires administered.

## Discussion

Our results indicate that the severity of anaphylaxis has implications for mental health, in particular regarding anxious symptoms. Anaphylactic patients with high severity of symptoms had a significant higher anxiety (HADS-A), a worse anaphylaxis-related quality of life (A-QoL-Adults), especially regard to the social impact of the anaphylaxis (SQoL), a significant lower physical well-being (Physical-WHOQoL BREF) and a particularly significant lower psychological well-being (Psychological-WHOQoL BREF), than patients with mild-moderate symptoms. On the other hand, the differences between high and mild-moderate symptoms in depression scores (HADS-D) were not significant, probably due to the small sample size. To these data are added important results on alexithymia. Few studies in the scientific literature have explored the presence of alexithymia in individuals with anaphylaxis, with most research conducted by Polloni et al. (13–15). In 2017, Polloni et al. found that, among food-allergic young patients, previous anaphylaxis and epinephrine prescription were associated with higher alexithymia scores, while age was inversely correlated, with younger patients exhibiting higher total alexithymia scores (13, 14). They also observed that individuals who had experienced anaphylaxis scored higher on the Difficulty Identifying Feelings (DIF) subscale than those who had not (14). In 2022, Polloni et al. reported that children with food allergies who had experienced anaphylaxis were more likely to be classified as alexithymic or borderline, with previous anaphylaxis significantly associated with higher DIF, Difficulty Describing Feelings (DDF), and total Toronto Alexithymia Scale-20 (TAS-20) scores (15).

In 2024, Ricciardi et al. investigated patients with severe allergic asthma or Hymenoptera venom anaphylaxis. They found that alexithymic patients or borderline alexithymic (38% of the total sample) exhibited higher levels of depression and anxiety, particularly in relation to psychological distress, and reported lower scores in vitality, social functioning, and mental health compared to non-alexithymic patients (16).

In the general population, alexithymia has a prevalence of approximately 10% (20). In Polloni et al. (2016), 55.3% of the sample was classified as borderline alexithymic using a TAS-20 score >51, while 38.2% were classified as alexithymic using the stricter cut-off of >60. In Ricciardi et al. (2019), 37.9% of patients were identified as borderline alexithymic with TAS-20 >51, and 25.6% with TAS-20 >60. Our study found 37.9% of patients with TAS-20 >51, which is directly comparable to the percentages reported in these studies using the same threshold.

These findings confirm existing scientific literature indicating that alexithymia is more prevalent in individuals with anaphylaxis than in the general population. Moreover, this study suggests that, among adults with anaphylaxis, alexithymia prevalence does not vary by age or gender. However, its prevalence remains lower in children with anaphylaxis.

The findings of this study indicate that individuals with alexithymia perceive the severity of anaphylaxis as significantly more distressing and disabling compared to non-alexithymic individuals. Specifically, the greater the difficulty in identifying, expressing, and describing emotions, the more severe the perception of anaphylaxis. Anaphylactic patients with alexithymia exhibited a higher tendency toward depression and a poorer perception of quality of life related to anaphylaxis, particularly in terms of the social impact of the condition and the limitations it imposes on daily life.

More severe alexithymia was associated with heightened anxiety and depressive symptoms, contributing to a progressively deteriorating perception of everyday life. This primarily affected interactions with the surrounding environment and psychological well-being, manifesting as diminished self-confidence, an increase in negative emotions, and difficulties in cognitive processes such as thinking and concentration.

In addition, an interesting result is that the EOT score had no statistically significant correlation with the other questionnaires administered. One possible explanation could be that the difficulty in recognizing their own emotions in alexithymic patients predisposes them to respond to the items on the emotional component of the anaphylaxis questionnaire (EOT) by underreporting or overreporting their emotional experiences, as would be expected from someone who experiences emotions in an undifferentiated and poorly regulated way. It could be useful to explore this hypothesis in future studies.

In summary, this study highlights the high prevalence of anxious symptoms in patients with more severe anaphylaxis and the significant negative impact of alexithymia on individuals with a history of anaphylaxis, profoundly shaping their experiences, particularly from psychological, environmental, and social perspectives. Therefore, beyond accurately classifying the condition, providing self-injectable epinephrine, and reassuring patients about their situation, it is crucial to focus on improving alexithymia when present. This requires appropriate and continuous psychotherapeutic support to help affected individuals identify, articulate, and describe their emotions. Such interventions could simultaneously enhance anxiety and depression levels, as well as the overall quality of life for individuals with a history of anaphylaxis.

In conclusion, our findings indicate that individuals with alexithymia not only perceive their anaphylactic episodes as more severe but also experience heightened emotional distress, as reflected in their elevated anxiety and depression scores. Furthermore, their lower environmental and psychological QoL scores underscore the necessity for tailored interventions that address both medical and psychological dimensions of patient care.

Integrating psychological assessments into anaphylaxis management allows healthcare providers to develop more comprehensive strategies beyond merely controlling physical symptoms. A holistic approach incorporating education, emotional support, and psychological interventions may enhance long-term outcomes for patients prescribed epinephrine auto-injectors.

This study has two primary limitations. The first pertains to the small sample size, as only 29 patients with anaphylaxis referred to the Allergology and Clinical Immunology clinic at Battipaglia Hospital agreed to participate. The study required responses to several psychological items, which often led to resistance and limited cooperation; some patients declined to provide their data, while others withdrew from the study. To enhance disease management, a critical future objective will be to educate patients on the significance of such questionnaires and provide support to help them overcome these barriers.

Additionally, this is a cross-sectional study in which questionnaires were administered only once during a single outpatient visit. Future longitudinal studies with larger samples could be highly beneficial in assessing potential improvements in the physical and psychological condition of patients with anaphylaxis, particularly after addressing alexithymia through appropriate psychological therapy.

Future research should further investigate these associations in larger populations to refine intervention models and optimize patient-centered care in anaphylaxis management.

## Data Availability

The raw data supporting the conclusions of this article will be made available by the authors, without undue reservation.

## References

[1] Warren C Dyer A Lombard L Dunn-Galvin A Gupta R. The psychosocial burden of food allergy among adults: a US population-based study. J Allergy Clin Immunol Pract. (2021) 9(6):2452–60.e3. 10.1016/j.jaip.2021.02.039 33677077 PMC9190169

[2] Lee Y Chang HY Kim SH Yang MS Koh YI Kang HR. A prospective observation of psychological distress in patients with anaphylaxis. Allergy Asthma Immunol Res. (2020) 12(3):496–506. 10.4168/aair.2020.12.3.496 32141262 PMC7061156

[3] Tal Y Shany G Hershko AY Ribak Y Mizrahi E Shamriz O. The association between anaphylaxis and post-traumatic stress disorder in subjects with hymenoptera venom allergy. J Allergy Clin Immunol Pract. (2020) 8(2):775–7. 10.1016/j.jaip.2019.07.035 31400478

[4] Casale TB Warren C Gupta S Schuldt R Wang R Iqbal A. The mental health burden of food allergies: insights from patients and their caregivers from the Food Allergy Research & Education (FARE) patient registry. World Allergy Organ J. (2024) 17(4):100891. 10.1016/j.waojou.2024.100891 38559493 PMC10973659

[5] Feng C Kim JH. Beyond avoidance: the psychosocial impact of food allergies. Clin Rev Allergy Immunol. (2019) 57(1):74–82. 10.1007/s12016-018-8708-x 30171460

[6] Pradhan S. Psychological burden of anaphylaxis and the fight for an EpiPen. BMJ Case Rep. (2021) 14(9):e243838. 10.1136/bcr-2021-243838 34479890 PMC8420693

[7] Jeong K Kim J Chang HY Song TW Kim JH Shin M. Maternal posttraumatic stress symptoms and psychological burden in mothers of Korean children with anaphylaxis. Allergy Asthma Immunol Res. (2022) 14(6):742–51. 10.4168/aair.2022.14.6.742 36426401 PMC9709689

[8] Santos AF Worm M Kurita S Wong T Contato D Pirillo E. Living with food allergies: the experiences of adult patients and caregivers. Front Allergy. (2023) 4:1272851. 10.3389/falgy.2023.1272851 38026132 PMC10658712

[9] Ciaccio C Dunne J Bever A Johnston K Kowal S Seetasith A. Living with and caring for people with multiple food allergies: a qualitative study. Patient Prefer Adherence. (2024) 18:1949–60. 10.2147/PPA.S466749 39318369 PMC11420886

[10] Warren C Gupta R Seetasith A Schuldt R Wang R Iqbal A. The clinical burden of food allergies: insights from the Food Allergy Research & Education (FARE) patient registry. World Allergy Organ J. (2024) 17(3):100889. 10.1016/j.waojou.2024.100889 38523669 PMC10959723

[11] Roleston C Protudjer JLP Herbert LJ Jones CJ Warren C Brough HA. “It's a permanent struggle to manage it really”: psychological burden and coping strategies of adults living with food allergy. Qual Health Res. (2025) 36(4-5):333–44. 10.1177/10497323251320839 40096850 PMC12982555

[12] Greiwe J. Quality of life and psychological issues associated with food allergy. J Food Allergy. (2023) 5(2):43–8. 10.2500/jfa.2023.5.230011 39022750 PMC11250200

[13] Polloni L Gregori D Ferruzza E Oricoli C Lazzarotto F Bonaguro R. Alexithymia in food-allergic versus healthy children and young adults. J Health Psychol. (2017) 22(2):228–36. 10.1177/1359105315600235 26349611

[14] Polloni L DunnGalvin A Ferruzza E Bonaguro R Lazzarotto F Toniolo A. Coping strategies, alexithymia and anxiety in young patients with food allergy. Allergy. (2017) 72(7):1054–60. 10.1111/all.13097 27886387

[15] Polloni L Ferruzza E Ronconi L D'Ovidio G Bonaguro R Lazzarotto F. Maternal anxiety and previous anaphylaxis are associated with alexithymia in young patients with food allergy. Pediatr Allergy Immunol. (2022) 33(1):e13680. 10.1111/pai.13680 34655502

[16] Ricciardi L Silvestro O Martino G Catalano A Vicario CM Lund-Jacobsen T. Health-related quality of life in severe hypersensitivity reactions: focus on severe allergic asthma and hymenoptera venom anaphylaxis-a cross-sectional study. Front Psychol. (2024) 15:1394954. 10.3389/fpsyg.2024.1394954 39246313 PMC11377323

[17] Nemiah JC Freyberger H Sifneos PE. Alexithymia: a view of the psychosomatic process. In: Hill OW, editor. Modern Trends in Psychosomatic Medicine. London: Butterworths (1976). Vol. 3; p. 430–9.

[18] Parker JDA Taylor GJ Bagby RM. Alexithymia and the recognition of facial expressions of emotion. Psychother Psychosom. (1993) 59:197–202. 8416096 10.1159/000288664

[19] Chiricozzi A Esposito M Gisondi P Valenti M Gori N Giovanardi G. Disease severity is associated with alexithymia in patients with atopic dermatitis. Dermatology. (2020) 236(4):329–35. 10.1159/000507246 32369808

[20] Holmes A Marella P Rodriguez C Glass Ii D Goerlich KS. Alexithymia and cutaneous disease morbidity: a systematic review. Dermatology. (2022) 238(6):1120–9. 10.1159/000524736 35636409

[21] Patella V Nettis E Peduto T Pierro L Pellacani G Bonzano L. Severe atopic dermatitis treated with anti-interleukin 4Rα reduces the psychological burden in patients with and without alexithymia. JAAD Int. (2023) 14:19–21. 10.1016/j.jdin.2023.07.020 38035127 PMC10682665

[22] Patella V Pierro L Bosco E Cuomo MS Florio G Latempa M. Anaphylaxis quality of life scale for adults: the validation on the Italian allergic patients. Eur Ann Allergy Clin Immunol. (2025). 10.23822/EurAnnACI.1764-1489.390 PMID: 40289815 40289815

[23] Knibb RC Huissoon AP Baretto R Ekbote A Onyango-Odera S Screti C. Development and validation of the anaphylaxis quality of life scale for adults. J Allergy Clin Immunol Pract. (2022) 10(6):1527–33.e3. 10.1016/j.jaip.2022.02.023 35259537

[24] The WHOQOL Group. Development of the World Health Organization WHOQOL-BREF quality of life assessment. Psychol Med. (1998) 28:551–8. 9626712 10.1017/s0033291798006667

[25] Skevington SM Lotfy M O'Connell KA. The World Health Organization's WHOQoL-BREF quality of life assessment: psychometric properties and results of the international field trial. A report from the WHOQoL Group. Qual Life Res. (2004) 13:299–310. 15085902 10.1023/B:QURE.0000018486.91360.00

[26] De Girolamo G Rucci P Scocco P Becchi A Coppa F D'Addario A. La valutazione della qualità della vita: validazione del WHOQOL-Breve [Quality of life assessment: validation of the Italian version of the WHOQOL-Brief]. Epidemiol Psichiatr Soc. (2000) 9(1):45–55. Italian. 10.1017/s1121189x00007740 10859875

[27] Zigmond AS Snaith RP. The hospital anxiety and depression scale. Acta Psychiatr Scand. (1983) 67:361–70. 6880820 10.1111/j.1600-0447.1983.tb09716.x

[28] Costantini M Musso M Viterbori P Bonci F Del Mastro L Garrone O. Detecting psychological distress in cancer patients: validity of the Italian version of the hospital anxiety and depression scale. Support Care Cancer. (1999) 7(3):121–7. 10.1007/s005200050241 10335929

[29] Cohen S Kamarck T Mermelstein R. A global measure of perceived stress. J Health Soc Behav. (1983) 24:385–96. 6668417

[30] Bagby RM Parker JD Taylor GJ. The twenty-item Toronto Alexithymia Scale–I. Item selection and cross-validation of the factor structure. J Psychosom Res. (1994) 38(1):23–32. 10.1016/0022-3999(94)90005-1 8126686

[31] Bagby RM Taylor GJ Parker JD. The twenty-item Toronto Alexithymia Scale–II. Convergent, discriminant, and concurrent validity. J Psychosom Res. (1994) 38(1):33–40. 10.1016/0022-3999(94)90006-x 8126688

